# Neighbourhood safety and area deprivation modify the associations between parkland and psychological distress in Sydney, Australia

**DOI:** 10.1186/1471-2458-13-422

**Published:** 2013-05-01

**Authors:** Shanley Chong, Elizabeth Lobb, Rabia Khan, Hisham Abu-Rayya, Roy Byun, Bin Jalaludin

**Affiliations:** 1 Centre for Research, Evidence Management and Surveillance, Sydney and South Western Sydney Local Health Districts, Locked Bag 7279 Liverpool BC, Sydney, NSW, 1871, Australia; 2 South Western Sydney Clinical School, University of New South Wales, Locked Bag 7017 Liverpool BC, Sydney, NSW, 1871, Australia; 3 School of Public Health and Community Medicine, University of New South Wales, Locked Bag 7017 Liverpool BC, Sydney, NSW, 1871, Australia; 4 Bureau of Health Information, Ministry of Health, Sydney, Australia; 5 School of Psychological Science, La Trobe University, Melbourne, Australia; and School of Psychology, The University of Sydney, Sydney, Australia

**Keywords:** Parklands, Greenspace, Mental health, Psychological distress, Area deprivation

## Abstract

**Background:**

The aim of this study was to investigate how perceived neighbourhood safety and area deprivation influenced the relationship between parklands and mental health.

**Methods:**

Information about psychological distress, perceptions of safety, demographic and socio-economic background at the individual level was extracted from New South Wales Population Health Survey. The proportion of a postcode that was parkland was used as a proxy measure for access to parklands and was calculated for each individual. Generalized Estimating Equations logistic regression analyses were performed to account for correlation between participants within postcodes, and with controls for socio-demographic characteristics and socio-economic status at the area level.

**Results:**

In areas where the residents reported perceiving their neighbourhood to be “safe” and controlling for area levels of socio-economic deprivation, there were no statistically significant associations between the proportion of parkland and high or very high psychological distress. In the most disadvantaged neighbourhoods which were perceived as unsafe by residents, those with greater proportions of parkland, over 20%, there was greater psychological distress, this association was statistically significant (20-40% parkland: OR=2.27, 95% CI=1.45-3.55; >40% parkland: OR=2.53, 95% CI=1.53-4.19).

**Conclusion:**

Our study indicates that perceptions of neighbourhood safety and area deprivation were statistically significant effect modifiers of the association between parkland and psychological distress.

## Background

Greenspace is defined as any vegetated land adjoining an urban area [1] and includes bushland, nature reserves, national parks, outdoor sports fields, school playgrounds and rural or semi-rural areas immediately adjoining an urban area. It can impact on physical and mental health through a number of pathways: it has a restorative function [2,3], provide opportunities for physical activity [4,5] and facilitate social connections [6,7]. Further, greenspaces and attractive surroundings can increase physical activity with its associated effects on physical and mental health. People who have better access to parks and greenspaces are more likely to report that they engage in physical activity [8-12] and attractive parks are associated with higher levels of walking [13]. However, there are also studies that report no associations between greenspace and physical activity [14,15] and hence the evidence for the impact of greenspace on physical activity is not as clear.

Parklands and greenspaces can also impact on well-being and mental health. Maas et al. [16] reported an association between the higher percentage of greenspace within a one or three kilometre radius and better self-rated health. De Vries et al. [17] also found positive associations between greenspace and self-rated health and that the effect was stronger in women who worked in the home and the elderly - the two groups of people who would be likely to spend more time in the local area. Grahn and Stigsdotter [18] reported associations between increased urban park use and lower levels of reported stress. In Australia, respondents who perceived their neighbourhood to be very green also reported better physical and mental health [19]. Further, greenspace can facilitate increased social connectedness [20-22] which in turn is associated with better health and well-being [23]. Here again the evidence for the beneficial effect of parkland on mental health and well-being is far from clear. For example, a study in England found no association between greenspace and self-rated health in higher income suburban and higher income rural areas and that greater amount of greenspace was associated with poorer health in low-income suburban areas [24]. Further, although van Dillen et al. showed a positive association between the quantity of greenspace and mental health, they were unable to demonstrate an association between the quality of greenspace and mental health[25].

Recent evidence suggests that neighbourhood physical and social characteristics, for example, vandalism, litter, safety, gangs and vacant housing are associated with psychological distress [26]. Even perceptions of violence, crime, drug use and graffiti are associated with mental health problems such as depression and anxiety [27]. Social factors such as neighbourhood deprivation, social isolation and social mobility are all associated with depression [28-31].

An important factor that may modify the effects of greenspace or parkland on physical activity and mental health is physical characteristics of the neighbourhood and the perception of how safe one feels in a neighbourhood. The perception that the neighbourhood is unsafe can lead people choosing not to use parks and greenspaces [32,33]. Furthermore, area deprivation may also mediate the effects of parkland on physical activity and well-being. The provision of parkland has been found to be poorer in lower socioeconomic status areas in one study [34]. The quality of parkland and greenspaces facilities and safety were poorer in more deprived areas [34,35] and residents living in deprived areas reported higher perceptions of problems with safety, more negative perceptions of their neighbourhood and were less likely to use the greenspaces [36,37]. Hence, perceptions of neighbourhood safety and area deprivation may be important factors in mediating the effects of parkland on mental health and well-being. No studies, as far as we aware, have investigated interactions between perceptions of neighbourhood safety, area deprivation and parkland, to explore how perceived neighbourhood safety and area deprivation change the relationship between parklands and mental health. Information from such studies could add to our understanding of the potential complex relationships between parklands and mental health and inform interventions to improve the overall health and well-being of residents. We therefore investigated associations between access to parkland and psychological distress in Sydney, Australia, and whether these associations were modified by perceptions of neighbourhood safety and area deprivation.

## Methods

The study area was limited to the Sydney Statistical Division and consists of 43 local government areas (LGAs) and 255 postcodes. The study area has a population of approximately 4.12 million people and covers an area of 12,428 square kilometres. The study was conducted in 2011.

### Access to parkland

We used the proportion of a postcode that was parkland as a proxy measure for access to parklands. We downloaded the 2006 land use dataset at the mesh block level from the Australian Bureau of Statistics website (http://www.abs.gov.au/AUSSTATS/abs@.nsf/DetailsPage/1209.0.55.0022006?OpenDocument). The mesh block is the smallest geographical unit, their boundaries cover the whole of Australia without gaps or overlaps and can be aggregated reasonably accurately to different geographical and administrative boundaries. We selected all mesh blocks in the study area that were identified as parkland. We allocated parkland mesh blocks to postcodes and calculated the proportion of the postcode that was parkland. Postcodes were then categorised into three groups: 0-19% parkland, 20-40% parkland and >40% parkland. These three categories were used in all analyses.

### Psychological distress and perception of neighbourhood safety

We obtained de-identified information about psychological distress, perceptions of safety and relevant covariates at the individual level from the 2007, 2008 and 2009 New South Wales (NSW) Population Health Surveys, NSW Ministry of Health. The NSW Population Health Survey is an on-going telephone survey of residents (from birth upwards conducted continuously between February and December each year). The target population for the surveys was all NSW residents living in households with private telephones and the target sample size was approximately 12,000 people per year. Households were contacted using list assisted random digit dialling. One person from the household was randomly selected for inclusion in the survey. Respondents were asked questions from modules on demographics, health behaviours, health status, and access to and satisfaction with health services.

There were 13,178 adult respondents in the 2007 NSW Population Health Survey, 10,296 adult respondents in the 2008 NSW Population Health Survey and 10,719 adult respondents in the 2009 NSW Population Health Survey, a total of 34,193 respondents aged 16 years or older. Of these respondents, 13,970 (41%) (5,443 from the 2007 survey, 4,241 from the 2008 survey and 4,286 from the 2009 survey) resided in metropolitan Sydney. Of the 13,970 respondents, 10,710 (77%) respondents had information on psychological distress.

Psychological distress was measured using the Kessler-10 (K10) instrument which is a 10-item questionnaire that measures the level of psychological distress in the most recent four week period [38]. A K10 score of ≥22 reflects high or very high psychological distress. Psychological distress was classified as high/very high and none/low/moderate. Perceptions of neighbourhood safety was derived from the following question: “My area has a reputation for being a safe place. Do you agree or disagree?” The responses were classified as strongly agree/agree and disagree/strongly disagree.

### Measurement of socio-economic status at the area level

The 2006 Index of Relative Socio-Economic Disadvantage (IRSED) at the postcode level was used in the analyses as a measure of area deprivation. The IRSED was created by the Australian Bureau of Statistics to compare social and economic disadvantage across geographical areas in Australia. The index is derived from the 2006 Census variables such as low income and educational attainment, high unemployment, and people working in unskilled occupations. The index has a mean score of 1,000 and standard deviation of 100 [39]. IRSED was categorised into tertiles for our analyses: tertile 1 (most disadvantaged group), tertile 2 (middle disadvantaged group) and tertile 3 (least disadvantaged group).

### Statistical methods

We used generalised estimating equations (GEE) logistic regression models to examine the association between parkland and high/very high psychological distress. GEE logistic regression accounted for correlation between participants within postcodes. As we had a large sample size, only statistically significant variables from the univariate regression analyses were included in the final model to account for any potential confounding effect. These variables were age, gender, household income, highest level of education completed, employment status, self-rated health and population density. Population density was included in the model as it can be associated with anxiety and depression in older people [40].

Both one-way and two-way interactions between safety, area deprivation index and parkland were explored. One-way interactions between perceived safety and area deprivation index, neighbourhood safety and parkland, and area deprivation index and parkland were all statistically significant (p<0.0001 for all three one-way interactions). The two-way interaction between safety, area deprivation index and parkland was also statistically significant (p<0.0001). The size of our dataset (n=10,710) provided sufficient statistical power to stratify the data rather using two-way interaction terms, which can be difficult to interpret. We therefore developed regression models, stratifying the data by reported neighbourhood safety.

The main effect of parkland on high/very high psychological distress generated from the GEE logistic regression model is presented in the Result section, followed by the GEE logistic regression model with interaction effects between area deprivation index and parkland.

Results from GEE models are presented as odds ratios (ORs) together with their 95 percent confidence intervals (95%CI). All estimates were weighted to adjust for differences in the probabilities of selection among subjects and for differences between the age and sex structure of the sample and Australian Bureau of Statistics mid-year population estimates for NSW. All analyses were conducted using Proc Genmod in SAS v9.2 (SAS Institute Inc., Cary, NC).

De-identified health survey data were provided to us by the NSW Ministry of Health who are the data custodians. The NSW Ministry of Health approved the use of the health survey data for this study. Ethics approval from an institutional human research ethics committee was not required.

## Results

The study area comprised 260 postcodes. The mean population of postcodes was 15,929 persons (standard deviation=12,839 persons), median was 13,147 persons and the range was 80–85,333 persons. The mean geographic area of postcodes was 56 km^2^, the median was 7.6 km^2^ and the range was 0.6-3,527 km^2^. The mean population density per postcode was 2,289 persons per square kilometre (standard deviation=1,824, median=1,938, range=1-11,442). There was a mean of 55 respondents per postcode (range=1-248).

Respondents who were females, younger, unemployed, had lower household incomes, who perceived their neighbourhood as not safe, lived in more disadvantaged areas and reported poor self-rated health were more likely to report high/very high psychological distress (Table 1). There were no statistically significant differences in the prevalence of reported high/very high psychological distress by the three categories of parkland (Table 1).

**Table 1:** **High or very high psychological distress by demographic characteristics** (**weighted to the population**)

	**High/very high psychological distress**^**1**^		
		**N=13,970**	**Univariate analysis**
**Gender**	**n**	%	OR. 95% CI
Male	5,486	7.2	0.71 (0.56−0.88)
Female	8,484	10.3	1
**Age group** (**years**)			
16−24	1,191	10.3	2.44 (1.69−3.54)
25−34	1,425	9.7	2.19 (1.51−3.18)
35−44	1,979	9.9	1.91 (1.38−2.65)
45−54	2,436	8.1	1.24 (0.93−1.67)
55−64	2,943	8.2	1.56 (1.16−2.11)
65+	3,996	5.4	1
**Household income**			
<$20,000	2,479	14.7	2.56 (1.83−3.58)
$20,000−$39,999	2,090	12.4	2.29 (1.67−3.15)
$40,000−$59,999	1,635	9.8	1.82 (1.28−2.59)
$60,000−$79,999	1,449	7.5	1.19 (0.78−1.82)
$80,000+	3,721	5.6	1
**Highest education**			
University	4,532	6.7	0.21 (0.06−0.74)
TAFE^2^	2,886	9.6	0.33 (0.10−1.14)
High school	2,330	8.4	0.31 (0.09−1.09)
Did not complete high school	4,026	11.4	0.38 (0.11−1.31)
Other	63	16.8	1
**Employment status**^3^			
Yes	7662	7.0	0.50 (0.40−0.62)
No	6268	12.2	1
**Index of Relative Socio-Economic Disadvantage**			
Most disadvantaged group	3,655	11.5	2.57 (1.79−3.69)
Middle disadvantaged group	7,943	8.5	2.13 (1.46–3.12)
Least disadvantaged group	2,371	5.0	1
**Perceived neighbourhood safety**			
Yes	6,916	7.2	0.47 (0.37−0.60)
No	2,376	14.5	1
**Self**-**rated health**			
Excellent, very good or good	10,282	6.3	0.25 (0.21−0.31)
Fair, poor or very poor	2,947	18.6	1
**Parkland**			
0−19%	6,659	8.9	1
20−40%	5,149	8.6	1.05 (0.76−1.43)
>40%	2,162	8.6	1.15 (0.78−1.70)

^1^The denominator includes yes, no and don’t know/refused. ^2^TAFE technical and further education certificate/diploma is a vocational tertiary qualification ranked below a university degree. ^3^Unemployment means did not have a job.

There were statistically significant associations between parkland and area deprivation (p<0.0001), between parkland and whether respondents thought their local area had a reputation for being a safe place (p=<0.0001) and area deprivation and perceptions of safety (p<0.0001).

In the main effects model, the associations between parkland and high/very high psychological distress was not statistically significant (20-40% parkland: OR=1.07, 95%CI=0.77-1.47, p=0.70; >40% parkland: OR=1.35, 95%CI=0.92-1.97, p=0.12). Respondents who perceived that their neighbourhood was not a safe place were generally more likely to report high/very high psychological distress if they lived in postcodes with ≥20% parkland compared to 0-19% parkland (Table 2). However, these associations were statistically significant only for those in the most disadvantaged areas. In the least disadvantaged areas, the magnitude of the associations between parkland and high/very high psychological distress, although non-statistically significant, were similar to those in the most disadvantaged areas. The exception was residents of the middle disadvantaged group who reported statistically significantly less psychological distress if they lived in postcodes with 20-40% parkland compared to 0-19% parkland.

**Table 2:** Associations between proportion of postcode that is parkland and high or very high psychological distress^*^

**Perception of neighbourhood safety**	**Area disadvantage (tertiles of IRSED)**	**Parklandx**	**Parkland**	**Parkland**
		**0−19%**	**20−40%**	**>40%**
		**OR**	**OR (95%CI)**	**OR (95%CI)**
			**p-value**	**p-value**
Not a safe neighbourhood	Most disadvantaged group	1	2.27 (1.45−3.55) <0.01	2.53 (1.53−4.19) <0.01
	Middle disadvantaged group	1	0.38 (0.15−0.92) 0.03	1.92 (0.95−3.88) 0.07
	Least disadvantaged group	1	2.15 (0.32−14.31) 0.43	2.92 (0.55−15.53) 0.21
Safe neighbourhood	Most disadvantaged group	1	1.02 (0.59−1.74) 0.96	1.21 (0.56−2.58) 0.63
	Middle disadvantaged group	1	0.81 (0.42−1.56) 0.53	0.68 (0.34−1.36) 0.28
	Least disadvantaged group	1	0.79 (0.39−1.59) 0.51	1.20 (0.55−2.59) 0.65

^*^Age, gender, household income, highest level of education completed, employment status and self-rated health included as individual level covariates in all models; population density included in all models as area level covariate.

There were no statistically significant associations between proportion of parkland and psychological distress in respondents who perceived that their neighbourhood was a safe place (Table 2). However, respondents in the middle group for area deprivation who perceived that their neighbourhood was not a safe place had non-statistically significant lower risks of high/very high psychological distress compared to residents in the most and least deprived postcodes.

## Discussion

In this study, there were no associations between the proportion of parkland and psychological distress where it was perceived that the neighbourhood was safe. However, there were significant statistically significant associations between the proportion of parkland and high or very high psychological distress when the proportion of parkland was ≥20% and if it was perceived that the neighbourhood was unsafe. As with many other studies, we controlled for important potential confounders such as age, gender, income, level of education and population density which, in our data, were associated with high or very high psychological distress. In addition, we also investigated interactions between proportion of parkland and perceptions of neighbourhood safety and area deprivation.

Although many studies have suggested that exposure to greenspace or parkland is associated with better health and well-being [16,17,19], we were not able to demonstrate that exposure to a higher proportion of parkland was associated with less psychological distress. However, we did find evidence indicating that perceptions of neighbourhood safety and area deprivation were statistically significant effect modifiers of the association between parkland and psychological distress.

No studies, as far as we are aware, have reported on effect modification of the association between parkland and mental health by area deprivation. Our findings show that increased psychological distress is associated with high proportion of parkland in both the most and the least socio-economically disadvantaged areas but only if the neighbourhood is perceived as unsafe. If the neighbourhood was perceived as safe, then there were only weak non-statistically significant associations between parkland and psychological distress regardless of area deprivation. In the United Kingdom, Mitchell and Popham [24] reported poorer self-rated health with increasing percentage of greenspace in suburban low income areas but not in urban and rural low income areas and suggest that this may be due to a larger proportion of poorer quality greenspace in low income suburban areas. Residents of deprived neighbourhoods also have poorer perceptions of access to greenspace (despite shorter mean distances to greenspace compared to less deprived neighbourhoods) [36,41] and safety of greenspace [36]. More disadvantaged areas are also more likely to have poorer lighting (Crawford et al.) which may influence safety from crime [11].

The importance of neighbourhood safety is also reflected in several papers where it is proposed that safe environments stimulate positive behaviours (e.g. amount of physical activity) and leads to reduction in stress [33,42,43]. Also, Agyemang et al. suggested feeling unsafe and dissatisfaction with greenspace was associated with poor self-rated health and discourages people engaging in outdoor activities [44]. Parkland associated with poor neighbourhood safety is consistently negatively associated with physical activity [41,45-47]. Jones et al. [36] reported statistically significant decreasing trends in levels of adequate physical activity with increasing perceptions of lack of safety of greenspace. Parklands may be a space for criminal activities and antisocial behaviours, and would explain why people may feel unsafe in neighbourhoods with large proportions of parkland [48]. This in turn may impact on recreational physical activity levels which in turn may adversely impact on psychological health and well-being.

In our study, respondents of the middle disadvantaged group, compared to the most disadvantaged and least disadvantaged areas, were less likely to report increased psychological distress with increasing percentages of parkland regardless of perceptions of the safety of the neighbourhood. It is not clear to us why this is so. This may be due to anomalies in the spatial level of our parkland data or to some important unmeasured confounders. However, it is not unusual to observe similar non-linear relationships in the literature. For example, a non-linear relationship between socio-economic status and psychological distress has been previously reported [49] and Poulos at al. [50] found a non-linear relationship between socio-economic status and childhood injuries.

The strengths of our study were that our subjects were part of an ongoing population based health status and risk behaviour survey, we had a large sample size and we took into account the multi-level nature of the data. The relatively high response rates (63.6% in 2007, 63.4% in 2008 and 58.7% in 2009) and the representativeness of the NSW Population Health Survey weighted sample ensures that our results are generalisable to metropolitan Sydney and other similar major Australian cities [51].

There are a number of limitations to our study. We obtained information on parkland from the ABS as land use data at the mesh block level. However, the electronic data did not allow us to analyse the data by number of discrete parks as a number of parcels of parkland could have contributed to a defined park. Further, parkland included state forests and national parks. We could not categorise parkland into more usable categories, for example, sports fields, bushland, presence of picnic facilities, etc., nor could we assess the quality of the parkland. We were also not able to calculate alternate metrics for access to parklands such as travel distance or travel time to parks as we did not have access to addresses for respondents in the NSW Health Surveys (addresses are not collected as part of the surveys). We were only able to obtain health survey data at the postcode level and hence we were restricted to using postcode as the unit of analysis. We would have preferred individual level data or data at smaller spatial units for analyses so as to have more accurate measures of exposure to parkland and to minimise exposure misclassification which is likely to be non-differential. This limitation of our study may decrease the precision of the estimates but not bias the results. Further, although the NSW Health Surveys were designed to provide estimates for spatial units larger than those used in this study, we do not expect this will affect our findings. Other limitations were that by using data from an existing cross-sectional survey we are not able to make a causal link between parkland and the reported health outcomes and that although we adjusted for a number of important potential confounders, there may yet be some residual confounding. However, we share this limitation with most other published studies on neighbourhoods and health.

## Conclusion

Our results highlight the importance of perceptions of neighbourhood safety and psychological distress and the findings should be noted by both government and planners. Further research should explore associations between park characteristics, different metrics for measuring access to parks (for example, distance to parks from residential address, work address and park), and their interaction with socio-demographic factors and neighbourhood environments. Such studies will require collaboration among a range of disciplines such as epidemiology, public health, psychology, urban planning, landscape design and transportation planning, to develop policies and recommendations beneficial to the overall health and well-being of residents.

## Competing interests

The authors declare that they have no competing interests.

## Authors’ contribution

SC, EL, RH and HAR conducted the study and drafted the manuscript. RB and BJ provided input into data interpretation and the draft manuscript. All authors read and approved the final version of the manuscript.

## Pre-publication history

The pre-publication history for this paper can be accessed here:

http://www.biomedcentral.com/1471-2458/13/422/prepub

## References

[B1] Scotland G Health impact assessment of greenspace: a guide 2008 Stirling: Edited by scotland g

[B2] Stigsdotter UK Ekholm O Schipperijn J Toftager M Kamper-Jørgensen F Randrup TB Health promoting outdoor environments - Associations between green space, and health, health-related quality of life and stress based on a Danish national representative survey Scand J Public Health 2010 38 4 411 417 10.1177/1403494810367468 20413584

[B3] Van Den Berg AE Maas J Verheij RA Groenewegen PP Green space as a buffer between stressful life events and health Soc Sci Med 2010 70 8 1203 1210 10.1016/j.socscimed.2010.01.002 20163905

[B4] Giles-Corti B Donovan RJ The relative influence of individual, social and physical environment determinants of physical activity Soc Sci Med 2002 54 1793 1812 10.1016/S0277-9536(01)00150-2 12113436

[B5] Wen M Kandula NR Lauderdale DS Walking for transportation or leisure: what difference does the neighborhood make? J Gen Intern Med 2007 22 12 1674 1680 10.1007/s11606-007-0400-4 17932724 PMC2219835

[B6] Abraham A Sommerhalder K Abel T Landscape and well-being: a scoping study on the health-promoting impact of outdoor environments Int J Public Health 2010 55 59 69 10.1007/s00038-009-0069-z 19768384

[B7] Morris N Health, well-being and open space 2003 Edinburgh: Edinburgh College of Art and Heriot-Watt University

[B8] Cohen DA Ashwood JS Scott MM Overton A Evenson KR Staten LK Porter D McKenzie TL Catellier D Public parks and physical activity among adolescent girls Pediatrics 2006 118 5 1381 1389 10.1542/peds.2006-1226 PMC2239262 17079539

[B9] Epstein LH Raja S Gold SS Paluch RA Pak Y Roemmich JN Reducing Sedentary Behavior Psychol Sci 2006 17 8 654 659 10.1111/j.1467-9280.2006.01761.x 16913945

[B10] Coombes E Jones AP Hillsdon M The relationship of physical activity and overweight with objectively measured green space accessibility and use Soc Sci Med 2010 70 6 816 822 10.1016/j.socscimed.2009.11.020 20060635 PMC3759315

[B11] McCormack GR Rock M Toohey AM Hignell D Characteristics of urban parks associated with park use and physical activity: a review of qualitative research Health Place 2010 16 712 726 10.1016/j.healthplace.2010.03.003 20356780

[B12] Kaczynski AT Henderson KA Environmental correlates of physical activity: a review of evidence about parks and recreation Leis Sci 2007 29 315 354 10.1080/01490400701394865

[B13] Giles-Corti B Broomhall MH Knuiman M Collins C Douglas K Ng K Lange A Donovan RJ Increasing walking: How important is distance to, attractiveness, and size of public open space? Am J Prev Med 2005 28 2, Supplement 2 169 176 15694525 10.1016/j.amepre.2004.10.018

[B14] Maas J Verheij R Spreeuwenberg P Groenewegen P Physical activity as a possible mechanism behind the relationship between green space and health: A multilevel analysis BMC Publ Health 2008 8 1 206 10.1186/1471-2458-8-206 PMC2438348 18544169

[B15] Wendel-Vos GCW Schuit AJ De Niet R Boshuizen HC Saris WHM Kromhout D Factors of the physical environment associated with walking and bicycling Med Sci Sport Exer 2004 36 725 730 10.1249/01.MSS.0000121955.03461.0A 15064601

[B16] Maas J Verheij RA Groenewegen PP de Vries S Spreeuwenberg P Green space, urbanity and health: how strong is the relation? J Epidemiol Community Health 2006 60 587 592 10.1136/jech.2005.043125 16790830 PMC2566234

[B17] de Vries S Verheij RA Groenewegen PP Spreeuwenberg P Natural environments-healthy environments? An exploratory analysis of the relationship between greenspace and health Environ Plan A 2003 35 1717 1731 10.1068/a35111

[B18] Grahn P Stigsdotter UA Landscape planning and stress Urban Forestry & Urban Greening 2003 2 1 18 10.1078/1618-8667-0001923545063

[B19] Sugiyama T Leslie E Giles-Corti B Owen N Associations of neighbourhood greenness with physical and mental health: do walking, social coherence and local social interaction explain the relationships? J Epidemiol Community Health 2008 62 5 e9 10.1136/jech.2007.064287 18431834

[B20] Coley RL Sullivan WC Kuo FE Where Does Community Grow? Environ Behav 1997 29 4 468 494 10.1177/001391659702900402

[B21] Kuo F Sullivan W Coley R Brunson L Fertile ground for community: Inner-city neighbourhood common spaces Am J Community Psychol 1998 26 6 823 851 10.1023/A:1022294028903

[B22] Kweon B-S Sullivan WC Wiley AR Green Common Spaces and the Social Integration of Inner-City Older Adults Environ Behav 1998 30 6 832 858 10.1177/001391659803000605

[B23] Kawachi I Berkman L Social ties and mental health J Urban Health 2001 78 3 458 467 10.1093/jurban/78.3.458 11564849 PMC3455910

[B24] Mitchell R Popham F Greenspace, urbanity and health: relationships in England J Epidemiol Community Health 2007 61 8 681 683 10.1136/jech.2006.053553 17630365 PMC2652991

[B25] van Dillen SME De Vries S Groenewegen P Greenspace in urban neighbourhoods and residents' health: adding quality to quantity J Epidemiol Commun Health 2011 66 8 1 5 10.1136/jech.2009.104695 21715445

[B26] Latkin CA Curry AD Hua W Davey MA Direct and indirect associations of neighbourhood disorder with drug use and high-risk sexual partners Am J Prev Med 2007 32 6S S234 S241 17543716 10.1016/j.amepre.2007.02.023 PMC1949488

[B27] Aneshensel CS Sucoff CA The neighbourhood context of adolescent mental health J Health Soc Behav 1996 37 4 293 310 10.2307/2137258 8997886

[B28] Mulvey A Gender, economic context, perceptions of safety, and quality of life: a case study of Lowell, Massachusetts (U.S.A.), 1982–96 Am J Community Psychol 2002 30 5 655 679 10.1023/A:1016321231618 12188055

[B29] Ross CE Reynolds JR Geis KJ The contingent meaning of neighbourhood stability for residents' psychological well-being Am Sociol Rev 2000 65 581 597 10.2307/2657384

[B30] Stockdale SE Wells KB Tang L Belin TR Zhang L Sherbourne CD The importance of social context: neighborhood stressors, stress-buffering mechanisms, and alcohol, drug, and mental health disorders Soc Sci Med 2007 65 9 1867 1881 10.1016/j.socscimed.2007.05.045 17614176 PMC2151971

[B31] Yen IH Kaplan GA Poverty area residence and changes in depression and perceived health status: evidence from the Alameda County Study Int J Epidemiol 1999 28 1 90 94 10.1093/ije/28.1.90 10195670

[B32] Seaman P Jones R Ellaway A It's not just about the park, it's about integration too: why people choose to use or not use urban greenspaces Int J Behav Nutr Phys Act 2010 7 1 78 10.1186/1479-5868-7-78 21029448 PMC2978120

[B33] Groenewegen PP van den Berg AE de Vries S Verheij RA: Vitamin G effects of green space on health, well-being, and social safety BMC Publ Health 2006 6 10.1186/1471-2458-6-149 PMC1513565 16759375

[B34] Estabrooks PA Lee RE Gyurcsik NC Resources for physical activity participation: does availability and accessibility differ by neighborhood socioeconomic status? Ann Behav Med 2003 25 2 100 104 10.1207/S15324796ABM2502_05 12704011

[B35] Crawford D Timperio A Giles-Corti B Ball K Hume C Roberts R Andrianopoulos N Salmon J Do features of public open spaces vary according to neighbourhood socio-economic status? Health Place 2008 14 4 889 893 10.1016/j.healthplace.2007.11.002 18086547

[B36] Jones A Hillsdon M Coombes E Greenspace access, use, and physical activity: understanding the effects of area deprivation Prev Med 2009 49 500 505 10.1016/j.ypmed.2009.10.012 19857513 PMC3748371

[B37] Ellaway A Macintyre S Bonnefoy X Graffiti, greenery, and obesity in adults: secondary analysis of European cross sectional survey Br Med J 2005 331 611 612 10.1136/bmj.38575.664549.F7 16113034 PMC1215553

[B38] Brooks RT Beard J Factor structure and interpretation of the K10 Psychol Assess 2006 18 62 70 16594813 10.1037/1040-3590.18.1.62

[B39] ABS Census of Population and Housing 2006 Canberra, Australia, ABS cat. no. 20330.0.55.001: Socio-Economic Indexes for Areas (SEIFA), Australia

[B40] Walker K Breeze E Wilkinson P GM. P, Bulpitt CJ, Fletcher A: Local area deprivation and urban–rural differences in anxiety and depression among people older than 75 years in Britain Am J Public Health 2004 94 10 1768 1774 10.2105/AJPH.94.10.1768 15451748 PMC1448532

[B41] Wilson DK Kirtland KA Ainsworth BE Addy CL Socioeconomic status and perceptions of access and safety for physical activity Ann Behav Med 2004 28 1 20 28 10.1207/s15324796abm2801_4 15249256

[B42] Ulrich RS Natural versus urban scenes: some psychophysiological effects Environ Behav 1981 13 523 556 10.1177/0013916581135001

[B43] Ulrich RS Simons RF Losito BD Fiorito E Stress recovery during exposure to natural and urban environments J Environ Psychol 1991 11 201 230 10.1016/S0272-4944(05)80184-7

[B44] Agyemang C van Hooijdonk C Wendel-Vos W Lindeman E Stronks K Droomers M The association of neighbourhood psychosocial stressors and self-rated health in Amsterdam, The Netherlands Journal of Epidemiological Community Health 2007 61 1042 1049 10.1136/jech.2006.052548 PMC2465673 18000125

[B45] Piro FN Noss O Claussen B Physical activity among elderly people in a city population: the influence of neighbourhood level violence and self perceived safety J Epidemiol Community Health 2006 60 7 626 632 10.1136/jech.2005.042697 16790836 PMC2566241

[B46] Gomez J Johnson BA Selva M Violent crime and outdoor physical activity among innner-city youth Prev Med 2004 39 876 881 10.1016/j.ypmed.2004.03.019 15475019

[B47] Molnar BE Gortmaker SL Bull FC Buka SL Unsafe to play? Neighborhood disorder and lack of safety predict reduced physical activity among urban children and adolescents Am J Health Promot 2004 18 5 378 386 10.4278/0890-1171-18.5.378 15163139

[B48] Ries AV Gittelsohn J Voorhees CC Roche KM Clifton KJ Astone NM The environment and urban adolescents' use of recreational facilities for physical activity: a qualitative study Am J Health Promot 2008 23 1 43 50 10.4278/ajhp.07043042 18785374

[B49] Phongsavan P Chey T Bauman A Brooks R Silove D Social capital, socio-economic status and psychological distress among Australian adults Soc Sci Med 2006 63 10 2546 2561 10.1016/j.socscimed.2006.06.021 16914244

[B50] Poulos R Hayen A Finch C Zwi A Area socioeconomic status and childhood injury morbidity in New South Wales Australia. Injury Prevention 2007 13 5 322 327 10.1136/ip.2007.015693 PMC2610604 17916889

[B51] NSWDOH New South Wales Population Health Survey 2010 Summary Report on Adult Health

